# Lack of Apoptosis of Infiltrating Cells as the Mechanism of High Susceptibility to EAE in DA Rats

**DOI:** 10.1155/2001/32636

**Published:** 2001

**Authors:** Miodrag L. Lukic, Eric Mensah-Brown, Sehamuddin Galadari, Allen Shahin

**Affiliations:** 1 Department of Medical Microbiology and Immunology Faculty of Medicine and Health Sciences UAE University PO Box 17666 Al Ain United Arab Emirates; 2 Department of Anatomy Faculty of Medicine and Health Sciences UAE University PO Box 17666 Al Ain United Arab Emirates; 3 Department of Biochemistry Faculty of Medicine and Health Sciences UAE University PO Box 17666 Al Ain United Arab Emirates

**Keywords:** EAE, cytokines, apoptosis, adjuvants

## Abstract

Dark Agouti (DA) rats are highly susceptible to induction of Th-l-mediated autoimmunity disease, including experimental allergic encephalomyelitis (EAE). In contrast to other susceptible rat strains in which disease is induced only with encephalitogen emulsified in complete Freund's adjuvants (CFA), in DA rats EAE develops after injection of encephalitogen in incomplete Freund's adjuvants (IFA) or Titermax, putative Th-2 directed adjuvant. Lymph node cells derived from immunized DA rats and stimulated *in* *vitro*  produce significantly more Interferon-γ (IFN-γ) than resistant Albino Oxford (AO) rats. However, cells derived from both strains produce large amounts of IL-10 but not IL-4. Immunized lymph node cells derived from EAE susceptible (AO × DA) F_1_rats induce clinical signs of disease in sublethally irradiated parental DA but not AO rats. The pathohistology of the target tissue in these recipients clearly demonstrated infiltration of mononuclear cells in both parental strains. However, the number of CD4^+^ cells was significantly higher and number of apoptotic cells significantly lower in DA rats sacrificed 8 days after passive transfer. We postulate that in addition to higher IFN-γ and TNF-α production, resistance to early apoptosis of the invading cells in the target tissue possibly due to lack of downregulation by TGF-β leads to exceptional susceptibility to EAE in DA rats.


*Developmental Immunology*, 2001, Vol. 8(3-4), pp. 193-200
Reprints available directly from the publisher
Photocopying permitted by license only

© 2001 OPA (Overseas Publishers Association)
N.V. Published by license under
the Harwood Academic Publishers imprint,
part of The Gordon and Breach Publishing Group,
a member of the Taylor and Francis Group

### Lack of Apoptosis of Infiltrating Cells as the Mechanism of High Susceptibility to EAE in DA Rats

MIODRAG L. LUKIC[a*], ERIC MENSAH-BROWN[b], SEHAMUDDIN GALADARI[c] and ALLEN SHAHIN[a]

[a]*Department of Medical Microbiology and Immunology,* [b]*Department of Anatomy and* [c]*Department of Biochemistry, Faculty of Medicine and Health Sciences, UAE University, PO Box 17666, Al Ain, UAE*

Dark Agouti (DA) rats are highly susceptible to induction of Th-1-mediated autoimmunity disease, including experimental allergic encephalomyelitis (EAE). In contrast to other susceptible rat strains in which disease is induced only with encephalitogen emulsified in complete Freund's adjuvants (CFA), in DA rats EAE develops after injection of encephalitogen in incomplete Freund's adjuvants (IFA) or Titermax, putative Th-2 directed adjuvant. Lymph node cells derived from immunized DA rats and stimulated *in vitro* produce significantly more Interferon-$\gamma$ (IFN-$\gamma$) than resistant Albino Oxford (AO) rats. However, cells derived from both strains produce large amounts of IL-10 but not IL-4. Immunized lymph node cells derived from EAE susceptible (AO $\times$ DA) $F_1$rats induce clinical signs of disease in sublethally irradiated parental DA but not AO rats. The pathohistology of the target tissue in these recipients clearly demonstrated infiltration of mononuclear cells in both parental strains. However, the number of $CD4^+$ cells was significantly higher and number of apoptotic cells significantly lower in DA rats sacrificed 8 days after passive transfer. We postulate that in addition to higher IFN-$\gamma$ and TNF-$\alpha$ production, resistance to early apoptosis of the invading cells in the target tissue possibly due to lack of downregulation by TGF-$\beta$ leads to exceptional susceptibility to EAE in DA rats.

*Keywords:* EAE, cytokines, apoptosis, adjuvants

### INTRODUCTION

T-helper type-1 (Th-1) cells appear to be involved in human organ specific autoimmune diseases. Multiple sclerosis is assumed to be a consequence of an autoimmune process in the central nervous system (CNS). Indeed, $CD4^+$ T cell clones derived from peripheral blood or cerebrospinal fluid of multiple sclerosis patients show a Th-1 lymphokine profile (Brod et al., 1991). Induction of experimental allergic encephalomyelitis (EAE) in susceptible mice and rats provides a remarkable model for the study of autoimmune inflammatory responses in CNS. Current evidence shows that autoreactive $CD4^+$ T cells cross the blood-brain barrier and secrete proinflammatory cytokines, IFN-$\gamma$ and TNF-$\alpha$, in response to epitopes present in CNS proteins such as myelin basic protein (MBP). We (Vukmanovic et al, 1990; Lukic et al., 1991) and others (Lorentzen et al., 1995; Lenz et al., 1999) have demonstrated that DA rats are exception-

* Address for Correspondence: Professor Miodrag L. Lukic, M.D., Ph.D. Department of Medical Microbiology Faculty of Medicine and Health Sciences UAE University PO Box 17666, Al Ain United Arab Emirates Tel: +971–3–7039514 Fax: +971–3–7671966 e-mail: m.lukic@uaeu.ac.ae ally susceptible to induction of EAE with MBP or encephalitogenic peptide, MBP 63–81, emulsified in CFA. Additionally, attempted tolerization of DA rats with MBP 63–81 peptide in IFA (Lenz et al., 1999) resulted in the induction of EAE. We have also shown that susceptible DA rats and (DA × AO) $F_1$ hybrids profoundly differ from EAE resistant AO rats in the level of production of Th-1 cytokines after appropriate stimulation. Significantly higher levels of Interleukin-2 (IL-2), IFN-$\gamma$ and TNF-$\alpha$ were produced by the stimulated spleen, lymph node and peritoneal cell populations of DA rats and (DA × AO) $F_1$ rats when compared to AO strain rats (Lukic et al., 1998).

Surprisingly, transfer of (AO × DA) $F_1$ encephalitogenic cells into sublethally irradiated parental hosts demonstrated that EAE develops only in DA rats (Mostarica-Stojkovic et al., 1992). Abundant information exists on the mechanisms involved in the induction phase of EAE such as immunodominant epitopes and effector lymphocyte phenotype but less is known about the factors responsible for the delivery of the signals which damage target cells. Although the molecular mechanisms are not understood, we have previously shown that TGF-$\beta$ downregulates EAE expression at the level of the target tissue (Shahin et al., 1995). Bonetti et al., (1997) have investigated expression of pro- and anti-apoptotic molecules in the CNS at different stages of EAE and found that expression correlated with elimination of infiltrating T cells and macrophages rather than oligodendrocytes.

In this study we have analyzed the extraordinary susceptibility to active and passive T cell mediated EAE in DA rats and resistance to EAE in AO rats at the level of the target tissue. We demonstrate that effector cells in DA rats are refractory to apoptosis at the level of CNS. We also observed that TNF-$\alpha$ inducible stress activated e-Jun-N-terminal kinase is more expressed in DA than in AO rat brain stem tissue 11 days after EAE induction. Taken together our data are compatible with the notion that, in the absence of downregulation at the level of the target tissue, possibly mediated by TGF-$\beta$, effector cells resist early apoptosis and induce TNF-$\alpha$ mediated pathology.

### MATERIAL AND METHODS

### Antigens and Adjuvants

Rat spinal cord or guinea pig MBP (Sigma, St Louis, USA) was used as the antigen, complete Freund's adjuvant (CFA) and copolymer CRL 89–41 (Titermax, Cytrx, USA) were used as adjuvants for EAE induction.

### Animals and Immunization

Eight to 12-week-old male DA and AO rats (originally purchased from Harlan, UK, and maintained in our animal facilities) were immunized in the left hind foot pad with antigen emulsified in adjuvant (0.1 ml of emulsion containing.005 ml of spinal cord tissue or 100 pg MBP). The animals were monitored for clinical signs of EAE, graded as 0 (no disease) 1 (loss of tail tonicity), 2 (hind limb weakness), 3 (hind limb paralysis), 4 (moribund or death), as previously described (Vukmanovic et al., 1990; Lenz et al., 1999).

### Cytokine Production

Draining lymph node cells were isolated from MBP + Titermax-primed rats 10 days after immunization and cultured for 4 days in the presence of MBP as detailed previously (Lukic et al., 1997). Supernatants from stimulated cells were assayed for IFN-$\gamma$, IL-4 and IL-10 using commercial ELISA kits (Biosource, USA).

### Passive Transfer

Passive transfer experiments were performed as detailed elsewhere (Mostarica-Stojkovic et al., 1992). (AO × DA) $F_1$ rats were immunized with spinal cord tissue emulsified in CFA. Ten days later draining lymph node cells were isolated and stimulated with MBP for 5 days. Sublethally irradiated (5 Gy) parental AO and DA rats and (AO × DA) $F_1$hybrids were i.v. injected with $1.5 \times 10^7$ cultured cells and animals sacrificed 8 days later. The spinal cord tissue was fixed in Zamboni's solution and embedded in paraffin.

### Immunostaining and detection of apoptosis

The spinal cord specimens were deparaffinized and incubated in 3.3% hydrogen peroxide in absolute methanol for 30 min to block endogenous peroxidase. The slides were then transferred into 0.01 M citrate buffer and boiled in a microwave oven for 5 min × 2 to retrieve antigens. The slides were washed in 0.05 M Tris-buffer, and incubated with a blocking agent containing carrier protein (Dako, Denmark). The blocking agent was poured off and the slides were incubated with mouse anti-rat monoclonal CD4 antibodies (Pharmingen, USA) (1 : 400, dilution in 0.05 M Tris-buffer) overnight at 4°C. The sections were allowed to warm to room temperature, washed three times (5 minutes each) in Tris-buffer (0.05 M Tris-BSA) and then incubated with the link antibody biotinylated anti-mouse IgG in PBS (DAKO, Denmark) for 30 minutes, followed up with avidin-peroxidase for 1 hour. The slides were then washed in 0.05 M Tris-buffer and peroxidase activity was demonstrated with diaminobenzidene hydrochloride (Sigma, USA; dissolved in 15 ml of M Tris-buffer, filtered, then 12 ml of 30% hydrogen peroxide added to the filtrate). The light brown solution was placed on the sections for 3–5 minutes. Sections were then counterstained within haematoxylin, sealed with a cover slip using Cytoseal 60 mounting medium (Stephens Scientific, USA) and examined on a Zeiss Axiophot photomicroscope.

The extent and localization of apoptosis *in situ* was assessed by direct immunoperoxidase detection of digoxigenin labelled genomic DNA in fixed tissue (Apoptag, *in situ* apoptosis detection kit, Oncor Inc., USA). The procedure recommended by the manufacturer was followed with counterstaining in methyl green.

### SDS-PAGE and Western Blotting

Animals were immunized with spinal cord tissue in CFA and groups of three animals sacrificed by day 11 and 17 after encephalitogen injection. Freshly isolated rat brain stem tissue was homogenized at 4°C in homogenization buffer (50 mM Hepes, 150 mM NaCI, 50 mM NaF, 1 mM $Na_3VO_4$, 10% glycerol, 10% Triton X-100, 1 μg/ml Leupeptin, 1 mM PMSF, pH 7.2). The homogenate was centrifuged at 15000 rpm for 15 minutes at 4°C. An aliquot of the supernatant was kept for protein determination, and to the rest, SOS sample buffer containing beta-mercaptoethanol was added. The samples were boiled for three minutes and after cooling, the equivalent of 30 μg of protein/lane were loaded on to a 12% polyacrylamide gel. The proteins were subjected to electrophoresis under constant voltage, transferred on to nitrocellulose membranes blocked with 50% non-fat milk. The blots were incubated with an antibody to JNK-1 (Santa Cruz Biotechnology, USA) at a dilution of 1 : 2000 at room temperature for 2 hours, washed and then incubated with the secondary antibody conjugated to horseradish peroxidase. Anti-JNK antibody binding was visualized by enhanced chemiluminescence.

### RESULTS

Given the extraordinary susceptibility of DA rats to develop EAE after the injection of encephalitogenic peptide in IFA adjuvant (Lenz et al., 1999), we tested whether an adjuvant that does not favour DTH responses such as Titermax (Bennett et al., 1992), would support induction of EAE. As shown in Table I, animals immunized with MBP emulsified in copolymer adjuvant did demonstrate clinical signs of disease by day 9 and developed severe EAE by day 11. In fact the disease was more severe than in DA rats challenged with MBP in CFA. As in previous experiments, AO rats were resistant to induction of disease with tested antigen in both adjuvants. Thus, our data demonstrate that the adjuvants that do not induce significant level of inflammation and apparently do not favour Th-1 responses, very effectively support activation of encephalitogenic T cells in DA rats.

TABLE I Induction of EAE in DA rats but not AO rats with myelin basic protein in CFA or Titermax

| *Strain* | *Immunogen* | *Adjuvant* | *Incidence* | *Onset (day)* | *Severity*[a] |
|---|---|---|---|---|---|
| DA | Spinal cord | CFA | 3/3 | 10.5 | 3.0 |
| | | Titermax | 6/6 | 9.0 | 3.5 |
| | MBP | MBP CFA | 3/3 | 11.5 | 2.6 |
| | | Titermax | 6/6 | 10.5 | 3.0 |
| AO | Spinal cord | CFA | 0/3 | | |
| | | Titermax | 0/6 | | |
| | MBP | CFA | 0/3 | | |
| | | Titermax | 0/3 | | |

a. Mean group severity (maximum = 4.0).

### Cytokine Production by Encephalitogen-Stimulated Lymph Node Cells

We have previously shown that lymphoid cells derived from DA rats produce significantly more IFN-γ in response to mitogens than corresponding population of AO rat-derived cells (Lukic et al., 1997). Additionally, LPS or IFN-γ stimulated macrophages produce significantly more TNF-α but not nitric oxide (NO) in DA rats (Lukic et al., 1998). To check whether these differences affect production of Th-1 cytokines by encephalitogen triggered lymph node cells, we analyzed production of IFN-γ, IL-4 and IL-10 in the cell populations restimulated *in vitro* with MBP. As illustrated in Table II, lymphoid cells in both strains produced significant amounts of IFN-γ. Although IL-4 was not detected in cells derived from both strains, IL-10 production was very high. These results imply that IL-10 does not efficiently downregulate expansion of encephalitogenic cells in DA rats.

### Passive Transfer of EAE

We have described previously (Mostarica-Stojkovic et al., 1992) that passive transfer of T cells derived from (AO × DA) $F_1$ hybrids do not induce clinical signs of disease in parental AO rats. In accordance with previous work, clinical signs of disease were seen in DA rats as early as day 6 after transfer (Table III). AO rats did not show any clinical signs of the disease. The recipient rats were sacrificed by day 8 after induction and sections of the spinal cords were examined for the presence of infiltrating cells. Haematoxylin eosin staining revealed mild perivascular infiltrates and diffusely infiltrating mononuclear cells in both AO and DA rats (data not shown). Although qualitative analysis was not attempted the number of infiltrating cells seemed to be similar in both strains. However, the number of $CD4^+$ cells appeared to be higher in DA strains (Fig. 1a and b). The most striking finding was the difference in the number of apoptotic cells in AO and DA CNS (Fig 1c and d). While most of the infiltrating cells in AO CNS were apoptotic, apoptosis was rarely seen by day 8 in the CNS of the DA rats. We concluded that resistance to apoptosis and expansion of disease inducing cells at the level of the target tissue significantly contribute to the extraordinary susceptibility of DA rats to EAE.

TABLE II *In vitro* Production of IFN-γ, IL-4 and IL-10 by the MBP-Titermax immunised lymph node cells re-stimulated with MBP[a]

| *Strain* | *Cylokincs (pg/ml)* | | |
|---|---|---|---|
| | *IFN-γ* | *IL-4* | *IL-10* |
| DA | 1120 | 0 | 846 |
| AO | 168 | 0 | 909 |

[a] $1 \times 10^6$ lymph node cells were cultured in the presence of 10 μg/ml MBP for 4 days.

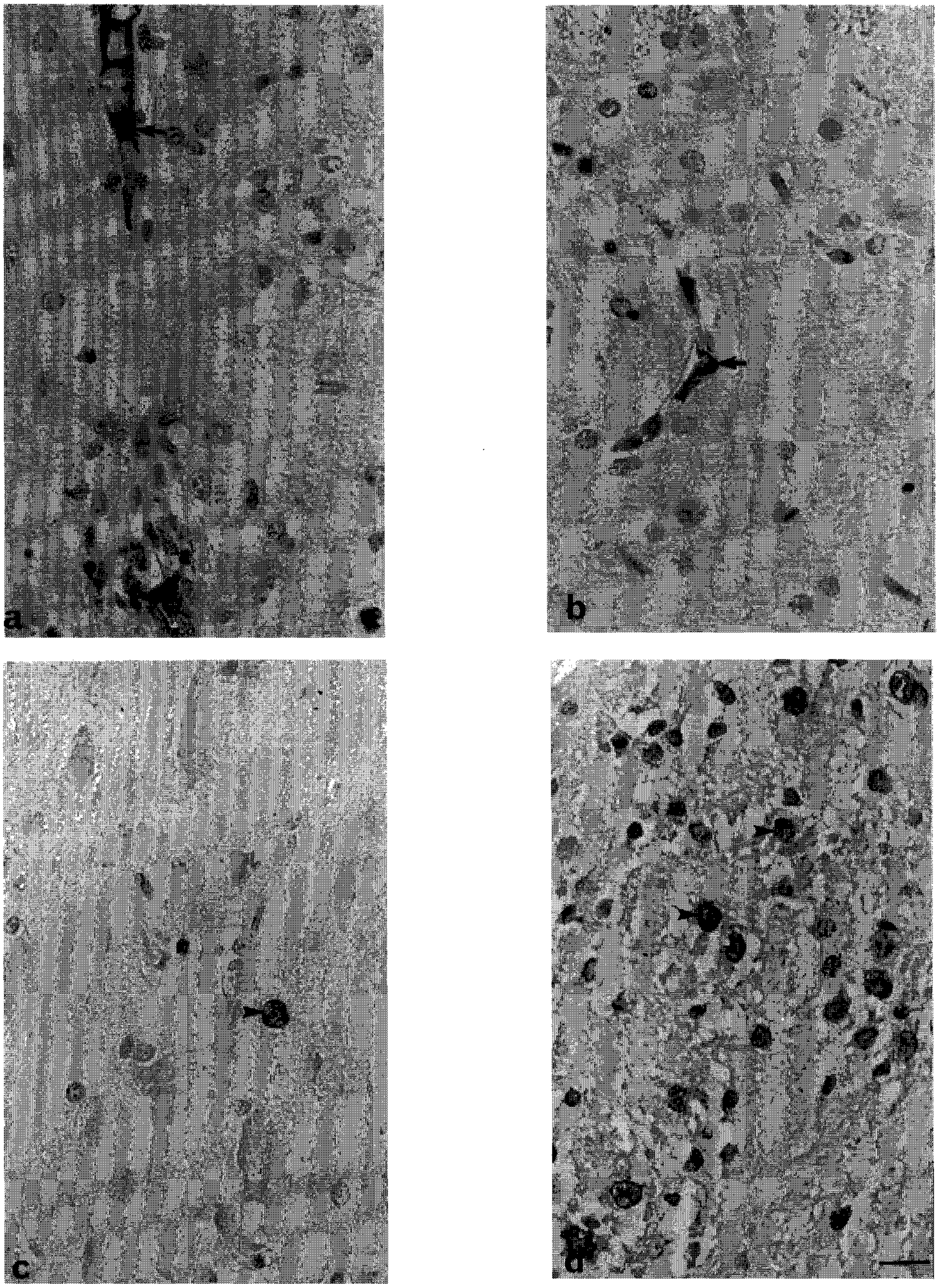

FIGURE 1 Light micrographs of cross sections of the spinal cord of DA (a, c) and AO (b, d) rats showing the CD4 immunoreactivity (arrows) in (a) DA and (b) AO rats and also a single apoptotic cell (arrowhead) in DA (c) and several apoptotic cells in AO (d). (Bar = 6 pm)

TABLE III Passive transfer of EAE[a]

| *Recipients* | *Incidence* | *nay of onset* | *Grade*[b] |
|---|---|---|---|
| DA | 3/3 | 6 | 1 |
| $F_1$ | 3/3 | 7 | 1,5 |
| AO | 0/5 | – | 0 |

a. $1.5 \times 10^7$ lymph node cells from (AO × DA) F1 rats immunized 10 days earlier with spinal cord tissue + Titermax and restimulated in vitrowith MBP for 5 days.
b. Mean group severity (Maximum = 4.0).

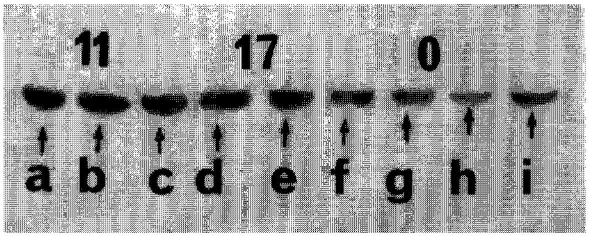

FIGURE 2 Western blot analysis of JNK expression in brain stem homogenate of AO, DA and F1 prior (g, h, I), and 11 (a, b, c) and 17 days (d, e, f) after EAE induction. The most pronounced increase of JNK expression was observed by day 11 in DA rats (h vs b)

### E-Jun-N-terminal kinases (JNKs) expression in the CNS after EAE induction

Expression of JNKs in the CNS may reflect transduction of a variety of stimuli to selective cellular response (Kumagae et al., 1999). Among the cytokines tested, TNF-α had the strongest effects of JNK expression in oligodendrocytes and astrocytes (Zhang et al., 1996). Given the fact that DA rats are "high" TNF-α producers in comparison to AO rats (Lukic et al., 1998) and that the brain stem was found to be target tissue in EAE in rats, we have analysed the level of JNK expression in the brain stem homogenate of AO and DA rats. The expression of JNKs was analysed at the peak of clinical disease in DA rats (day 11) and by 17 days after the disease induction. These data are illustrated in Fig 2. It was found that JNK expression is lower in untreated DA rats but when compared with AO and (AO × DA) $F_1$, the increase of expression is highest in DA rats by day 11 after active immunization.

### DISCUSSION

The main findings of the studies reported in here are the following: (a) DA rats develop EAE following immunization with MBP when given with an adjuvant that does not contain mycobacteria (Table I); (b) this regimen induces high IFN-γ production in the presence of IL-10 producing Th-2 cells (Table II); (c) susceptibility to passive EAE in DA rats correlated with lack of apoptosis in the target tissue when compared with EAE resistant AO rats (Fig. 1); (d) susceptibility to EAE correlated with enhanced expression of JNK stress activated protein kinase (Fig. 2).

Our early work in DA and AO rats (Mostarica-Stojkovic et al, 1992; Vukmanovic et al. 1990) and that of Lenz et al., (1999) demonstrated that DA rats are highly susceptible to EAE. Nonionic block copolymer adjuvants, such as Titermax, are reported to induce high, long lasting antibody titers to soluble proteins without inducing local granulomatous reaction (Bennett et al., 1992). Preliminary experiments also indicated that Titermax does not support induction of delayed type hypersensitivity (manufacturer's report 1999). However, in DA rats both "tolerogenic regimen", such as encephalitogenic peptide in IFA (Lenz et al., 1999), as well as encephalitogen in putative Th-2 directed adjuvants, led to induction of EAE (Table I). These findings challenge the paradigm that immunization protocols which favour Th-2 cells in some strains of EAE susceptible mice and rats are inevitably tolerogenic (Forsthuber et al., 1996). Instead, it appears that genetics of host may override the effects of adjuvant relative to induction of autoaggressive T cell responses. Interestingly, it was found that in addition to disease promoting IFN-γ, lymph node cells stimulated with MBP + Titermax produced significant amounts of IL-10. This implies that at least in DA rats, IL-10 may not be an immunoregulatory cytokine suppressing expansion of encephalitogenic cells (Table II). Indeed, IL-10 has been produced by encephalitogenic T cell lines not only in DA (Lenz et al., 1999) but also in Lewis rats (Sun et al., 1995). In order to further understand the strain differences in susceptibility to EAE, we analyzed the events at the level of the target tissue. To equalize the influx in

CNS, we used sublethally irradiated parental strains and encephalitogenic $F_1$ (AO × DA) cells. This protocol has been already established and has demonstrated that only DA rats exhibit clinical signs of the disease (Mostorica-Stojkovic et al., 1992). It had been demonstrated that while molecules of the apoptotic cascade are well represented in the CNS during EAE, their expression correlates with elimination of infiltrating cells (Bonetti et al., 1997; White et al., 1998). The selective apoptotic elimination of autoreactive T cells from the target organ of spontaneously resolving EAE has been also reported after passive transfer of disease in Lewis rats (Tabi et al., 1994). To this, we now add the evidence that early apoptosis of infiltrating cells may contribute to the lack of clinical signs of EAE in resistant AO rats. It has been reported recently that Fas/FasL interactions play a role in the pathogenesis of EAE, but that they are not required for disease to occur (Dittel et al., 1999). On the other hand, extraordinary susceptibility to EAE in DA rats may be due to the presence of costimulatory signal in the CNS, which upregulates the expression of anti-apoptotic molecules. Although expression of these proteins may be modulated by cellular interaction in CNS (van Parijs et al., 1996; White et al., 1998) the precise mechanism of survival of $CD4^+$ T cells in DA CNS is not understood. One of the possible explanations is the absence of downregulation mediated to TGF-β induced apoptosis. We have shown previously that TGF-β downregulats EAE in DA rats (Shahin et al., 1995). The production of IFN-γ and TNF-α by (DA × AO) $F_1$ encephalitogenic cells may then lead to initial damage of oligodendrocytes and initiate demyelination. Indeed demyelinisation has been shown to be mediated by TNF-α (Selmaj and Raine, 1998) and we have shown that peritoneal macrophages derived from DA rats are "high" TNF-α producers (Lukic et al., 1998). To this we now add the evidence that the expression of JNK protein kinase highly sensitive to TNF-α activation (Zhang et al., 1996) is more significantly enhanced after EAE induction in DA rats than in EAE resistant AO rats.

Taken together, our data support the notion that expression of organ-specific autoimmunity may be regulated at the level of the target tissue. It appears that susceptibility of DA rats to organ-specific autoimmunity is not related only to preferential activation of Th-1 disease inducing cells but also to relative lack of downregulatory Th-2 related, apoptosis inducing mechanisms, at the level of the target tissue.

### *Acknowledgements*

Critical reading by Dr S Dissanayake, secretarial assistance of Mr MV Raghavan and support by an FMHS UAE University grant are gratefully acknowledged.

### *References*

Bennett B., Check I.J., Olsen M.R., and Hunter R.L. (1992). A comparison of commercially available adjuvants for use in research. J Immunol Methods **153** 31–40.

Bonetti B., Pohl J., Gao Y-L., and Raine C.S. (1997). Cell death during autoimmune demyelination: Effector but not target cells are eliminated by apoptosis. J Immunol **159**: 5733–5741.

Brod S.A., Benjamin D., and Hafler D.A. (1991). Restricted T cell expression of IL-2, IFN-γ mRNA in human inflammatory disease. J Immunol **147**: 810–815.

Dittel B.N., Merchant R.M., and Janeway C.A. Jr. (1999). Evidence for Fas-dependent and Fas-independent mechanisms in the pathogenesis of experimental autoimmune encephalomyelitis. J Immunol **162**: 6392–6400.

Forsthuber T., Yip H.C., and Lehmann P.V. (1996). Induction of Th1 and Th2 immunity in neonatal mice. Science **271**: 1728–1731.

Kumagae Y., Zhang Y., Kim O.J., and Miller C.A. (1999). Human c-Jun N-terminal kinase expression and activation in the nervous system. Brain Res Mol Brain Res. **67(1)**: 10–7.

Lenz D.C., Wolf N.A., and Swanborg R.H. (1999). Strain variation in autoimmunity: Attempted tolerization of DA rats results in the induction of experimental autoimmune encephalomyelitis. J Immunol **163**: 1763–1768.

Lorentzen J.C., Issazadeh S., Storch M., Mustafa M.I., Lassman H., Linington C., Klareskog L., and Olsson T. (1995). Protracted, relapsing and demyelinating experimental autoimmune encephalomyelitis in DA rats immunized with syngeneic spinal cord and incomplete adjuvant. J Neuroimmunol **63**: 193–198.

Lukic M.L., Ejdus L., Pravica V., Shahin A., Stosic S., Mostarica M., Liew F.W., Ramic Z., and Badovinac V. (1997). Downregulation of Th-1 mediated autoimmune pathology. In Immunoregulation in Health and Disease, Lukic et al., Ed. (London/New York: Academic Press), pp 265–278.

Lukic M.L., Mostarica Stojkovic M., Vukmanovic S., Ramic Z., Petrovic M., and Ejdus L. (1991). Requirements for the induction of experimental allergic encephalomyelitis: Controlling mechanisms in the inductive phase and at the level of the target tissue. Period Biol **93**: 105–111.

Lukic M.L., Stosic-Grujicic S., and Shahin A. (1998). Effector mechanisms in multiple low-dose streptozotocin induced diabetes. Dev Immunol **6**: 119–128.

Mostarica-Stojkovic M., Vukmanovic S., Ramic Z., and Lukic M.L. (1992). Evidence for target tissue regulation of the resistance to experimental allergic encephalomyelitis in AO rats. J Neuroimmunol **41**: 99–106.

Selmaj K.W., and Raine C.S. (1998). Tumor necrosis factor mediates myelin and oligodendrocyte damage in vitro. Ann Neurol **23**: 339–347.

Shahin A., Mahmoud T.A.M., and Lukic M.L. (1995). Transforming growth factor $\beta$ and Interferon $\gamma$ modulate the development of Th-1 mediated autoimmunity in susceptible and resistant rats. Transpl Proc **27**: 1535–1537.

Sun D., Hu X-z., Shah R., and Coleclough C. (1995). The pattern of cytokine gene expression induced in rat T cells specific for myelin basic protein depends on the type and quality of antigenic stimulus. Cell Immunol **166**: 1–8.

Tabi Z., McCombe P.A., and Pender M.P. (1994). Apoptotic elimination of $V\beta8.^{2+}$ cells from the central nervous system during recovery from experimental autoimmune encephalomyelitis induced by the passive transfer of $V\beta8.2^{+}$ encephalitogenic T cells. Eur J Immunol **24**: 2609–2617.

van Parijs L., Ibraghimov A., and Abbas A.K. (1996). The roles of costimulation and Fas in T cell apoptosis and peripheral tolerance. Immunity **4**: 321–328.

Vukmanovic S., Mostarica Stojkovic M., Calud I., Ramic Z., and Lukic M.L. (1990). Analysis of T cell subsets after induction of experimental autoimmune encephalomyelitis in susceptible and resistant strains of rats. J Neuroimmunol **27**: 63–69.

White C.A., McCombe P.A., and Pender M.P. (1998). The roles of Fas, Fas ligand and Bcl-2 in T cell apoptosis in the central nervous system in experimental autoimmune encephalomyelitis. J Neuroimmunol **82**: 47–55.

Zhang P., Miller B.S., Rosenzweig S.A., and Bhat N.R. (1996). Activation of C-jun N-terminal kinase/stress-activated protein kinase in primary glial cultures. J Neurosci Res **46(1)**: 114–21.

